# Localization of *Daucus carota* NMCP1 to the nuclear periphery: the role of the N-terminal region and an NLS-linked sequence motif, RYNLRR, in the tail domain

**DOI:** 10.3389/fpls.2014.00062

**Published:** 2014-02-26

**Authors:** Yuta Kimura, Kaien Fujino, Kana Ogawa, Kiyoshi Masuda

**Affiliations:** ^1^Laboratory of Plant Functional Biology, Chair of Botany and Agronomy, Graduate School of Agriculture, Hokkaido UniversityHokkaido, Japan; ^2^Laboratory of Crop Physiology, Chair of Botany and Agronomy, Graduate School of Agriculture, Hokkaido UniversityHokkaido, Japan

**Keywords:** NMCP1, nuclear localization signal (NLS), nuclear envelope, *Daucus carota*, lamin, GFP-fusion protein, site-directed mutation

## Abstract

Recent ultrastructural studies revealed that a structure similar to the vertebrate nuclear lamina exists in the nuclei of higher plants. However, plant genomes lack genes for lamins and intermediate-type filament proteins, and this suggests that plant-specific nuclear coiled-coil proteins make up the lamina-like structure in plants. NMCP1 is a protein, first identified in *Daucus carota* cells, that localizes exclusively to the nuclear periphery in interphase cells. It has a tripartite structure comprised of head, rod, and tail domains, and includes putative nuclear localization signal (NLS) motifs. We identified the functional NLS of DcNMCP1 (carrot NMCP1) and determined the protein regions required for localizing to the nuclear periphery using EGFP-fused constructs transiently expressed in *Apium graveolens* epidermal cells. Transcription was driven under a CaMV35S promoter, and the genes were introduced into the epidermal cells by a DNA-coated microprojectile delivery system. Of the NLS motifs, KRRRK and RRHK in the tail domain were highly functional for nuclear localization. Addition of the N-terminal 141 amino acids from DcNMCP1 shifted the localization of a region including these NLSs from the entire nucleus to the nuclear periphery. Using this same construct, the replacement of amino acids in RRHK or its preceding sequence, YNL, with alanine residues abolished localization to the nuclear periphery, while replacement of KRRRK did not affect localization. The sequence R/Q/HYNLRR/H, including YNL and the first part of the sequence of RRHK, is evolutionarily conserved in a subclass of NMCP1 sequences from many plant species. These results show that NMCP1 localizes to the nuclear periphery by a combined action of a sequence composed of R/Q/HYNLRR/H, NLS, and the N-terminal region including the head and a portion of the rod domain, suggesting that more than one binding site is implicated in localization of NMCP1.

## Introduction

The nuclear envelope (NE) is a structure that lies between the nucleoplasm and cytoplasm. In metazoans, the NE is composed of the inner nuclear membranes, outer nuclear membranes, nuclear pore complex (NPC), and nuclear lamina. The nuclear lamina is a proteinaceous meshwork composed mainly of the type V intermediate filament proteins, the nuclear lamins, which line the inner surface of the nuclear membranes. In addition to their role in diffusing local mechanical stress on the NE, lamins are involved in many aspects of nuclear function (Gruenbaum et al., 2003, 2005; Dahl et al., 2004; Starr and Fischer, 2005; Dechat et al., 2010). Defects in lamins lead to abnormal nuclear morphology and, in humans, cause physiological disease resulting in developmental disorders and premature aging (Broers et al., 2006).

Lamin genes can be identified in all metazoans, and it is conceivable that the ancestral gene has been retained in the animal kingdom with evolution (Dechat et al., 2010). Lamins have not been identified in lower eukaryotes (Goldman et al., 2002; Melcer et al., 2007). Likewise, complete sequencing of several plant genomes and systematic analyses of coiled-coil proteins, including theoretical translation products, have revealed that plants lack genes for lamin and its relatives (Mewes et al., 1998; Rose et al., 2004; Meier, 2007). In higher plants, electron micrographs of cells from which the NE membranes and chromatin have been removed manifest an electron-dense structure at the periphery of residual nuclei (Moreno Díaz de la Espina et al., 1991; Masuda et al., 1993). This structure possesses similar traits to the lamina in terms of resistance to chemical extraction (Moreno Díaz de la Espina et al., 1991; Ciska et al., 2013). Recently, using a field emission scanning electron microscope, observations of a filamentous lattice attached to the inner nuclear membrane and a nonrandom array of the NPC suggested anchoring to the filamentous architecture in BY-2 cells (Fiserova et al., 2009). The lattice was named a plamina, representing the plant lamina (Fiserova et al., 2009; Fiserova and Goldberg, 2010).

NMCP1, a plant protein originally identified in *Daucus carota* cells (Masuda et al., 1997), localizes exclusively at the nuclear periphery. Like lamins, it has a tripartite structure composed of central coiled-coils (rod domain) and nonhelical terminal regions (head and tail domains) having theoretical NLS (nuclear localization signal) motifs. NMCP1 homologues have been characterized in such plants as *Arabidopsis thaliana, Oryza sativa*, and *Allium cepa* (Moriguchi et al., 2005; Dittmer et al., 2007; Ciska et al., 2013). *A. graveolens* NMCP1 and 2 (AgNMCP1 and AgNMCP2) localize at the nuclear periphery in interphase cells but lose their integration during mitosis (Kimura et al., 2010). They dissociate almost simultaneously at prometaphase with NE breakdown. Then, type 1 AgNMCP becomes distributed around the mitotic spindles and accumulates on the surface of segregating chromosomes, while type 2 becomes distributed in the mitotic cytoplasm and accumulates at the periphery of decondensing chromosomes after segregation has been completed (Kimura et al., 2010). There are four DcNMCP1-homologues, LINC1 through LINC4, in *Arabidopsis*, and a double mutant of LINC1 and 2 produce small plants with small nuclei (Dittmer et al., 2007). LINC1 through LINC3 and LINC4 are grouped into two different families containing type 1 NMCP and type 2 NMCP, respectively (Ciska et al., 2013). LINC1 and LINC4 play predominant roles in the maintenance of nuclear morphology in leaf and root epidermal cells of *Arabidopsis* (Sakamoto and Takagi, 2013).

*De novo* synthesized NMCP1/LINC1 localizes at the nuclear periphery (Moriguchi et al., 2005; Dittmer et al., 2007); however, the mechanism of localization is not clear. Lamins A and B target the NE membrane with the aid of isoprenylation of their carboxy-terminal sequences (Holtz et al., 1989; Kitten and Nigg, 1991), and interact more stably with integral NE proteins such as LBR (lamin B receptor) and LAP1 and 2 (lamin-associated proteins 1 and 2) (Worman et al., 1988; Foisner and Gerace, 1993). Still, no motif that specifies isoprenylation has been found in the NMCP members. The amino-terminal region of human LBR fused with green fluorescent protein (GFP) is targeted to the NE in BY2 cells ectopically expressing the protein (Irons et al., 2003; Graumann et al., 2007), though the target recognized by that portion of LBR remains to be identified. Sad1-UNC-84 homologous (SUN) proteins that reside at the inner nuclear membrane interact with the lamina at its N-terminal region (Tapley and Starr, 2012). SUN proteins associate with Klarsicht, ANC-1, Syne Homology (KASH) proteins to form a bridge from the karyoskeleton to the cytoskeleton (Starr and Fischer, 2005). *A. thaliana* SUN-domain proteins (AtSUNs) contain an evolutionarily conserved transmembrane domain at the C-terminal region and localize at the inner nuclear membrane (Graumann et al., 2010). They associate with AtWIPs (plant-specific proteins with KASH functions) and have been suggested to link to LINC1 and LINC2 (Zhou et al., 2012). AtSUNs are reported to control the morphology and position of the nucleus (Oda and Fukuda, 2011). Myosin XI-i residing on the outer nuclear membrane links to the cytoskeleton and interacts with AtWIPs, and their linkage controls nuclear movement and shape in *Arabidopsis* (Tamura et al., 2013). AtSUN-YFP expressed in stably transformed BY-2 cells have been used as markers for investigating the dynamics of the post-breakdown NE membranes (Graumann and Evans, 2011), and to examine the applicability of the endoplasmic reticulum (ER) retention model (Anderson et al., 2009; Guttinger et al., 2009) to plant systems (Graumann and Evans, 2011).

Localization of NMCP1 may be caused by anchoring to the NE and/or peripheral chromatin. It is conceivable that localization is closely associated with the function of NMCP1, and defining protein regions responsible for localization is a practical approach to elucidating their functions. In this study, using enhanced GFP (EGFP) fused with a partial sequence from DcNMCP1, and employing an efficient plant system to detect the expressed fusion proteins, we determined the functional NLS and deduced the protein regions responsible for localization of DcNMCP1 to the nuclear periphery.

## Results

### Functional NLSs of DcNMCP1

**Figure 1:**
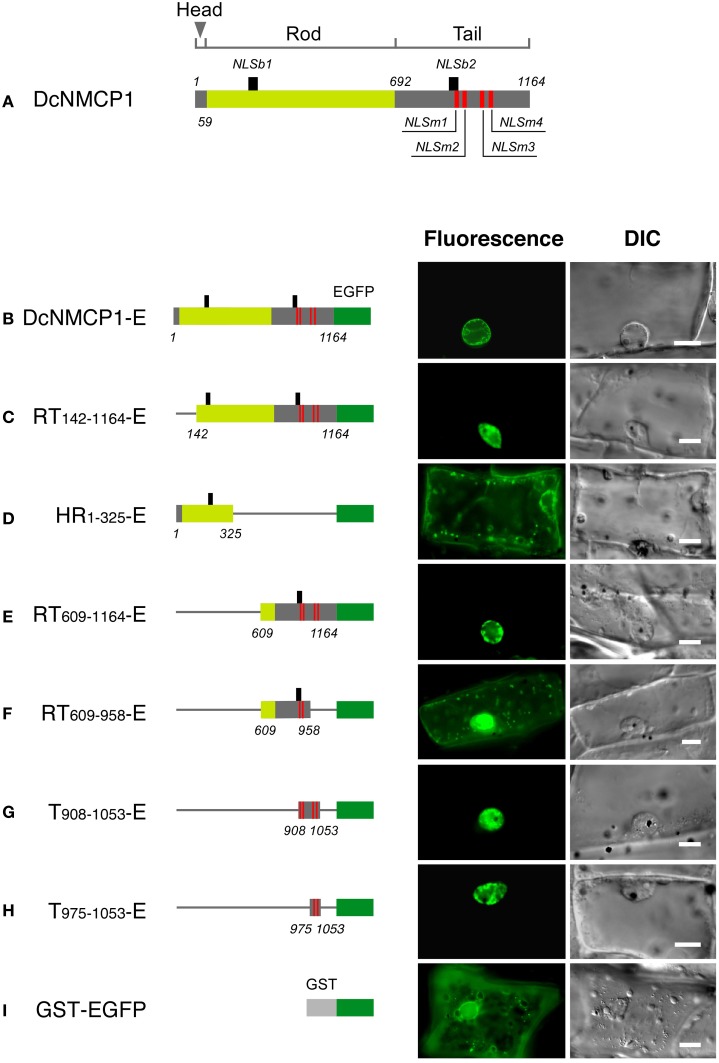
**The region of DcNMCP1 that functions to localize to the nucleus includes two NLS motifs. (A)** Position of the putative NLS motifs in DcNMCP1. The SV-40 large T-antigen type and bipartite type NLS motifs are indicated by red and black vertical bars, respectively. The head (amino acids 1–58), rod (amino acids 59–69), and tail (amino acids 692–1164) domains are represented by different colors. Localization of the full length **(B)** and deletion constructs of DcNMCP1 **(C–I)** fused with EGFP were expressed in *A. graveolens* epidermal cells. Constructs are schematically represented on the left of each micrograph. Thin rods indicate the deleted region. Captured fluorescence microscopy images were processed based on the 2D blind deconvolution algorithm. Bars = 10 μm.

Transiently expressed EGFP-fused full-length DcNMCP1 (DcNMCP1-E) showed clear fluorescence at the nuclear periphery (Figure 1B). EGFP fused with GST showed a high level of fluorescence in the cytoplasm (Figure 1I). Frequently, fluorescence of GST-EGFP was intense around the nucleus, perhaps due to stacked ER membranes and a dense cytoplasm.

In DcNMCP1, five SV-40 large T-antigen type NLS motifs (called classical NLSs) were identified at amino acids 908–914, 917–920, 1004–1008 (motifs at 1004–1007 and 1005–1008 overlapped), and 1023–1026 (Figure 1A), provisionally named NLSm1, NLSm2, NLSm3, and NLSm4, respectively (Figure 1A). Another NLS motif consisting of two short stretches of basic residues separated by a short spacer (bipartite NLS) was identified at amino acids 197–213 and 906–922; they were named NLSb1 and NLSb2. NLSb1 was found in the rod domain, while other NLSs resided in the tail domain.

The construct containing all of the NLS motifs localized exclusively to the nucleus (Figures 1B,C). The region including only NLSb1 did not show appreciable localizing activity (Figure 1D), and the region including all of the NLS motifs except NLSb1 (RT_609−1164_-E) localized to the nucleus (Figure 1E). The deletion construct RT_609−958_-E, which contained NLSm1, NLSm2, and NLSb2, showed considerable fluorescence in the cytoplasm and very weak localization to the nucleus (Figure 1F). A region including NLSm3 and NLSm4 (T_975−1053_-E) exclusively localized to the nucleus, and no appreciable fluorescence was detected in the cytoplasm (Figure 1H). It showed a distribution pattern similar to that of the construct containing all classical NLS motifs (compare Figures 1E,G,H). Thus, the nuclear localizing activity of DcNMCP1 was attributable to NLSm3 and NLSm4. NLS m1 and NLSm2 were only weakly active and the bipartite-type NLS motifs were inactive.

### Limited regions are required for localization to the nuclear periphery

**Figure 2:**
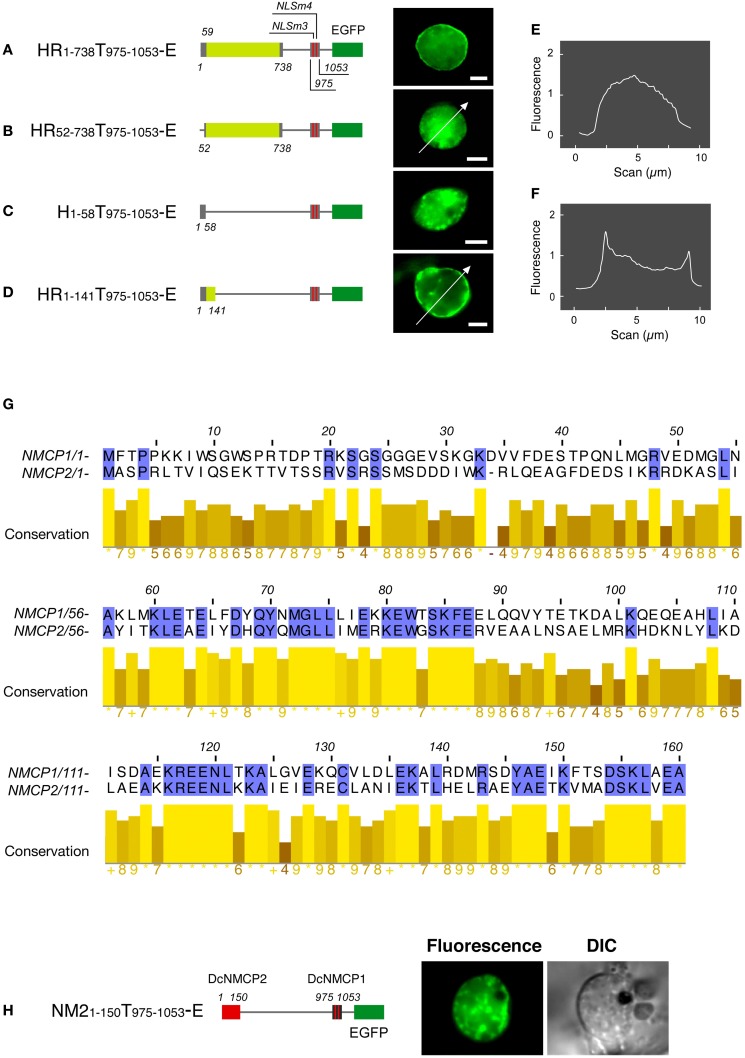
**The N-terminal region is required for the DcNMCP1 to localize to the nuclear periphery.** Identification of the region required for localization to the nuclear periphery **(A–D)**. Constructs of fusions are schematically represented in the left of micrographs. The micrographs show EGFP-fluorescence of *A. graveolens* cells expressing fusion proteins, and the fluorescence intensity distribution along the lines indicated in **(B)** and **(D)** are represented in **(E)** and **(F)**. The fluorescence intensity was analyzed using ImageJ 1.46r software (http://rsbweb.nih.gov/ij/index.html). **(G)** Sequence similarity of the N-terminal regions between NMCP1 and NMCP2. **(H)** Effect of replacement of the first 141 amino acids in HR_1-141_T_975 - 1053_-E by the amino acids 1–150 region of DcNMCP2 on localization to the nuclear periphery. Bars = 5 μm.

**Figure 3:**
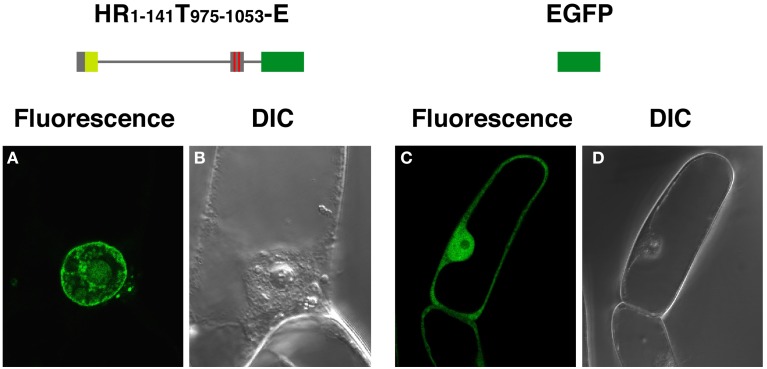
**HR_1-141_T_908-1053_-E expressed in BY-2 cells localizes to the nuclear periphery.** BY-2 cells were transformed by an *Agrobacterium*-mediated transformation method, using a binary vector pBI121. EGFP-fluorescence from HR_1-141_T_908–1053_-E **(A)** and EGFP **(C)**, were examined under a confocal laser scanning microscope. **(B)** and **(D)** show corresponding DIC images.

Fusions having the N-terminal region (amino acids 1–738) of DcNMCP1 combined with a portion of the tail domain that includes functional NLSs (amino acids 975–1053) localized to the nuclear periphery (Figure 2A). Deletion of the 111 C-terminal residues of DcNMCP1, which contain an evolutionarily conserved 11 amino acid sequence at the C-terminus, did not affect localization. The construct with the 141 N-terminal residues deleted, RT_142−1164_-E, failed to localize to the nuclear periphery (see Figure 1C). The effect of deletion was brought about by eliminating only the N-terminal 51 residues (HR_52−738_T_975−1053_-E) (Figures 2B,E), but the addition of the N-terminal 58 residues to the sequence at 975–1053 (HR_1−58_T_975−1053_-E) did not restore localization (Figure 2C). Addition of a longer region (amino acids 1–141) to the sequence of amino acids 975–1053 permitted localization to the nuclear periphery (Figures 2D,F), similarly to the addition of amino acids 1–738 (Figure 2A). Localization of expressed HR_1−141_T_975−1053_-E to the nuclear periphery was examined in stably transformed BY-2 cells. Extensive fluorescence of expressed HR_1−141_T_975−1053_-E was found at the nuclear periphery, although slight distribution in the nucleoplasm was also detected (Figure 3).

**Figure 4:**
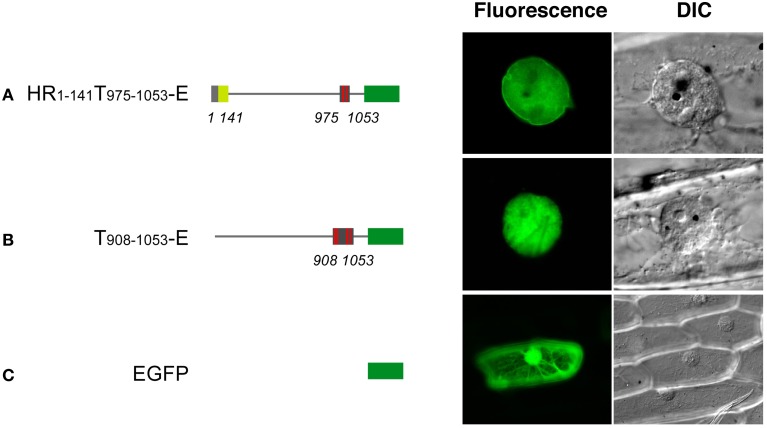
**Transiently expressed HR1-141T908–1053-E localizes to the nuclear periphery in *Allium cepa* epidermal cells.** Vectors including genes for HR1-141T908–1053-E **(A)**, T908–1053-E **(B)** and EGFP **(C)** were introduced into the epidermal tissue by a DNA-coated microprojectile delivery system. Constructs of fusions are schematically represented in the left of micrographs.

Expression of EGFP-fused full-length DcNMCP1 was scarcely detectable in *A. cepa* epidermal tissue. A deletion construct, HR_1−58_T_975−1053_-E, however, showed a similar localization pattern to that in *A. graveolens* cells (Figure 4). The discrepancy may be attributable to the difference in nuclear size and/or expression activity between the two recipient cell systems.

The regions between amino acids 60–160 of DcNMCP1 and 59–159 of DcNMCP2 show significant sequence similarity (Figure 2G). Accordingly, the N-terminal region of DcNMCP1 was replaced with the corresponding NMCP2 region in order to compare the effect of the head domain on localization to the nuclear periphery. Unexpectedly, the chimeric construct containing amino acids 1–150 from DcNMCP2 fused with amino acids 975–1053 of DcNMCP1 did not localize to the nuclear periphery (Figure 2H).

### The conserved sequence R/Q/HYNLRR/H functions in localization to the nuclear periphery

**Figure 5:**
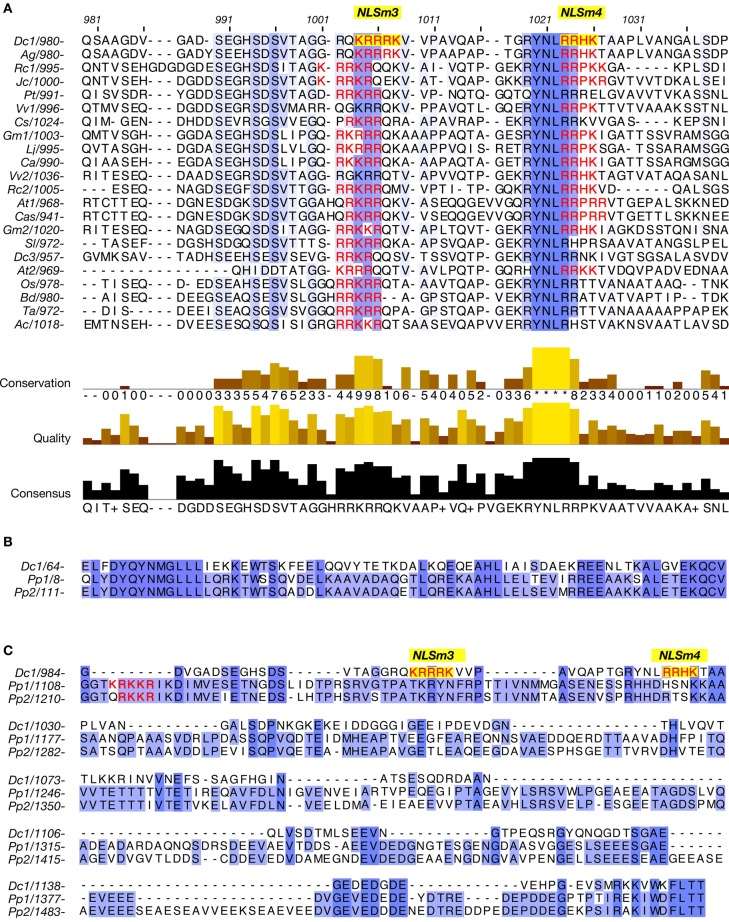
**A region required for localization to the nuclear periphery includes the conserved amino acid sequence R/Q/HYNLRR/H. (A)** Alignment of a region including functional NLSs of DcNMCP1 and the corresponding sequence of homologous proteins from other plants. **(B,C)** Alignment of regions from DcNMCP1 and NMCP1-like proteins deduced from *Physcomitrella patens* hypothetical genes. These homologues include the consensus MGLLL and a carboxy-terminal amino acid sequences but lack the sequence motif R/Q/HYNLRR/H. NLS motifs are indicated by red characters (NLSm3 and NLSm4 are indicated by yellow boxes). Plant species: Dc1 (NMCP1) and Dc3 (NMCP3) (*Daucus carota*), Ag (*Apium graveolens*), Cs (*Cucumis sativus*), At1 (LINC1) and At2 (LINC2) (*Arabidopsis thaliana*), Rc1 and Rc2 (*Ricinus communis*), Gm1 and Gm2 (*Glycine max*), Lj (*Lotus japonicus*), Jc (*Jatropha curcas*), Ca (*Cicer arietinum*), Cas (*Camelina sativa*), Sl (*Solanum lycopersicum*), Pt (P*opulus trichocarpa*), Vv1 and Vv2 (*Vitis vinifera*), Os (*Oryza sativa*), Bd (*Brachypodium distachyon*), Ta (*Triticum aestivum*), Ac (*Allium cepa*), Pp1 and Pp2 (*Physcomitrella patens*). Accession numbers and other tags of the sequences are listed in Supplemental table S3.

Alignment of amino acids 975–1053 of DcNMCP1 with corresponding regions from plant homologues indicated that these regions contain an evolutionarily conserved sequence, R/QYNLRR/H (R, arginine; Q, glutamine; Y, tyrosine; N, asparagine; L, leucine; H, histidine) at amino acid positions 1020–1025 in DcNMCP1 (Figure 5A). The last two amino acids, RR, frequently overlap with NLSm4. In other cases, the NLS motif was found less than 20 amino acids from the beginning of the sequence R/Q/HYNLRR/H (Figure 5A). NMCP1 homologues deduced from moss (*Physcomitrella patens*) hypothetical genes lack this motif (Figures 5B,C).

**Figure 6:**
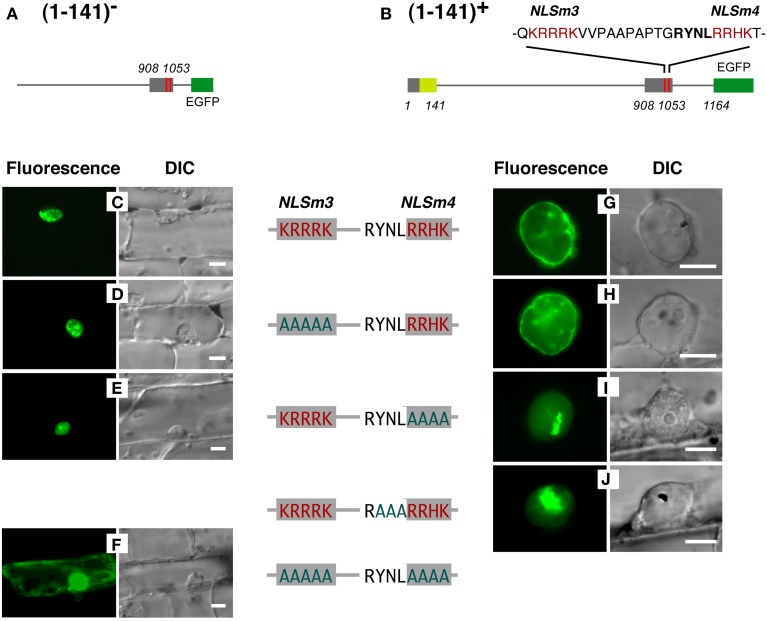
**Replacement of amino acids in YNL and/or RRHK with (A) cancels localization of a DcNMCP1-derived protein region to the nuclear periphery.** Effects of amino acid replacements were examined, using constructs with **(G–J)** or without **(C–F)** the N-terminal 141 amino acids (constructs shown in **A,B**). Substituted residues in the sequence are indicated by red characters and original residues are indicated in greenish-blue. Bars = 10 μm.

The NLS activity of NLSm3 and NLSm4, and the function of the RYNLRR sequence for localization to the nuclear periphery were then examined by site-directed mutagenesis of amino acid residues. Amino acid replacement with A (alanine) in either NLSm3 or NLSm4 in T908–1053-E (Figure 6A) did not affect the nuclear localization of the fusion proteins (Figures 6D,E), whereas replacement in both NLSs negated nuclear localization ability (Figure 6F), indicating that both NLSm3 and NLSm4 act as the NLS.

When the N-terminal 141 amino acids from DcNMCP1 were added to T908–1053 (Figure 6B), localization was modified, leading to accumulation at the nuclear periphery (Figures 6C,G). Amino acid replacement of NLSm3 in this construct with A did not affect localization to the nuclear periphery (Figure 6H), whereas the replacement of NLSm4 by A abolished localization to the nuclear periphery (Figure 6I); localization in the nucleus was unaffected by this manipulation. Moreover, the replacement of YNL (amino acids 1020–1022), immediately preceding NLSm4, with A also abolished localization to the nuclear periphery (Figure 6J).

Proteins that lost the ability to localize to the nuclear periphery frequently emitted fluorescence as large foci in the nucleoplasm (Figures 6I,J). These artificial foci were found only when constructs included the N-terminal region of DcNMCP1, indicating that the functional loss of the R/QYNLRR sequence causes irrelevant association of the N-terminal region.

## Discussion

As elucidated in this work, expressed DcNMCP1 localizes to the nuclear periphery by coordinate action of an N-terminal region, the NLS, and an NLS-linked hexapeptide sequence motif. Two NLS motifs in the tail domain are involved in translocation into the nucleus, and the hexapeptide R/QYNLRR/H, linked to the functional NLS, has a unique role in allowing incorporated DcNMCP1 to localize to the nuclear periphery in coordination with the action of the N-terminal region.

This hexapeptide motif is found in NMCP1 across a wide range of plant species, and includes the consensus sequence RYNLR (Ciska et al., 2013), though variants such as *A. thaliana* LINC3 (NM_105552) and homologues deduced from *P. patens* hypothetical genes lack this motif; LINC3 includes a similar sequence RYQLR. On the other hand, AgNMCP2 (NMCP2 of *A. graveolens*) localizes to the nuclear periphery of interphase cells, despite lacking the R/Q/HYNLRR/H sequence. *A. graveolens* NMCP2 is distributed in the mitotic cytoplasm after NE breakdown and accumulates at the nuclear periphery after the assembly of NMCP1 (Kimura et al., 2010); accumulation of AgNMCP2 occurs during the end of cell division and appears to continue after the cell plate is formed. Its dynamics show a clear difference from that of AgNMCP1. Thus, the NMCP family can be divided into three classes. NMCP1 members are divided into two classes, based on whether or not it carries the motif R/QYNLRR/H. The third class includes AgNMCP2 and its homologues, which are phylogenetically distant from the other two classes. These variations imply that NMCP is a rapidly growing family that participates in diverse nuclear processes.

Of the predicted NLS motifs in DcNMCP1, only two SV-40 large T-antigen type NLSs were highly functional. One appears to be implicated in localization to the nuclear periphery; the sequence YNLR in DcNMCP1 overlaps with a functional NLS, RRHK. YNLR-carrying NMCP1 in monocots and some dicots lacks an NLS motif at the overlapping position, though in these plants, the NLS motif is necessarily found at a short distance (~16 amino acids) from the beginning of the consensus sequence, R/Q/HYNLRR/H. The short distance between R/Q/HYNLRR/H and the NLS suggests their close association is required for localization to the nuclear periphery.

When connected to the 141 N-terminal amino acids from DcNMCP1, the tail domain derived from DcNMCP1 localizes to the nuclear periphery. The N-terminal region is likely to control coiled-coil dimerization and enable association with existing architecture at the nuclear periphery. Algorithmic prediction indicated that the coiled coil begins 12 amino acids before the beginning of the consensus sequence, MGLLL at amino acids 72–76, and hence, the N-terminal 141 amino acid region of DcNMCP1 forms dimers through short coiled coils. Accordingly, elimination of the first 51 amino acids and/or the following amino acids 52–141 from DcNMCP1 is presumed to lead to impeded dimerization or subsequent organization. The crucial role of the head domain in lamins for polymerization has been shown in *in vitro* experiments using recombinant proteins; dimers made of wild-type lamin B_2_ associate longitudinally to form polar head-to-tail polymers (Strelkov et al., 2004), whereas headless chicken lamin B_2_ dimers lose the propensity to form the polar association (Heitlinger et al., 1992; Stuurman et al., 1998; Isobe et al., 2007). In contrast, tailless lamins are able to polymerize longitudinally by polar head-to-tail association (Heitlinger et al., 1992).

The number of amino acids that form the rod domains is nearly constant in NMCPs from various plants, implying that they are organized into a highly ordered, stable structure through lateral association *in vivo*. The region including the first 141 amino acids of DcNMCP1 functions in localization to the nuclear periphery, suggesting that the rod domain in the full-length protein is dispensable for localization. Nevertheless, the stability of the lateral association of NMCP1 and association with the NE must be critical for building the peripheral architecture. It is necessary to examine whether constructs lacking a major part of the rod domain can reside stably at the nuclear periphery, and whether the conformational distance between the N-terminal region and the sequence R/Q/HYNLRR/H is significant for the stability of localization.

Unexpectedly, the 141 N-terminal amino acid region of DcNMCP1 cannot be replaced with the corresponding region of DcNMCP2, though these regions include the top of the rod domain and share consensus sequences. This result implies that the N-terminal region of NMCP1 have unique traits. NMCP1 and 2 do differ in accumulation patterns during NE formation, suggesting that they are incorporated into different architectures at the nuclear periphery, just as filaments made from A-type and B-type lamins build different structures and differ in organization in the lamina (Goldberg et al., 2008). Chimeric DcNMCP1 and 2 might not properly associate with the structural domains of the nucleus and cause failure in localization to the nuclear periphery.

Although open mitosis evolved independently in the plant and animal lineages, they appear to share similar NE dynamics (Rose, 2008). Some aspects of NMCP1 are amazingly similar to lamins, despite the lack of domains specific for the intermediate-type filament protein family. It has been pointed out that intermediate filament proteins may have evolved during the transition from a closed to an open mitosis in the animal lineage (Cohen et al., 2001; Goldman et al., 2002; Taddei et al., 2004). Likewise, the NMCP gene family may have evolved in the plant lineage along with the development of open mitosis. It is conceivable that the structural and functional similarities between NMCPs and lamins arose convergently during the development of mitotic systems.

## Methods and materials

### Transient gene expression in *Apium graveolens* cells

For transient gene expression, expression vectors for the EGFP fusions were affixed to gold particles (#165–2264, Bio-Rad, http://www.bio-rad.com) and introduced into plant cells from 3 cm above peeled epidermal tissues at 1100 psi, using a PDS-1000/He (Bio-Rad) microparticle delivery system.

The expression of full-length DcNMCP1-EGFP was first examined using epidermal tissues of *A. cepa* scales; however, the fluorescence was too weak to evaluate localization. Thus, we tested tissues from several plant species and selected epidermal tissues from *A. graveolens* (celery) petioles. The dorsal epidermis of outer petioles was peeled with fine forceps after cutting into approximately 5 × 30 mm squares. The peeled epidermis is made up of tissue with single- and partially double-layered cells. The tissue piece was placed, peeled surface upward, on semi-solid medium containing a half-strength inorganic salt mixture from Murashige and Skoog medium for particle bombardment. Transformed tissues were incubated for 16 h at 25°C in the dark. Tests for each expression vector with different constructs were performed at least three times.

### Vector construction for transient expression

Vectors pJB1414 and pKD0330, which carry the CaMV35S promoter followed by the EGFP-coding sequence and a nopaline synthase terminator, were used. DNA fragments encoding full and partial sequence for DcNMCP1 were inserted into the *Stu*I site between the promoter and the EGFP-coding sequences after introducing a *Stu*I site by PCR, which generated plasmids expressing fusion proteins with C-terminal EGFP. The sequences of the primers used for PCR are shown in Supplemental tables S1, S2. In most cases, to ensure the normal conformation of EGFP, reverse primers were designed to insert a spacer of 3–5 glycine residues before EGFP.

Expression vectors for a single EGFP-fused protein that included two discrete regions of DcNMCP1 were made as follows. A cDNA fragment of a region close to the C-terminus of DcNMCP1 was extended to include the additional sequence 5^′-CCT-3^′^^ at the 5^′^ end and a repeated 5^′^-GGTGGA-3^′^ sequence at the 3^′^ end for glycine repeats. The fragment with the extensions was then inserted into the blunt-end *Stu*I restriction site of pKD0330 to yield EGFP at the C-terminus. A cDNA fragment encoding another DcNMCP1 sequence was then inserted into the newly formed *Stu*I cutting site before the previous insert. Similarly, a cDNA fragment encoding the N-terminal region of DcNMCP2 (Accession number: AB514508) was inserted into the vectors to express a fusion protein with a partial DcNMCP1-EGFP sequence.

### Microscopy and image processing

Transient gene expression in epidermal tissues from *A. graveolens* petioles and *A.cepa* scales were examined using an Olympus BX50 fluorescence microscope (http://www.olympus.co.jp/jp/) equipped with UPlanApo (100×) and UPlan Fl (40×, 20×) objectives and DIC optics. Images were captured using a C4742 CCD camera (Hamamatsu Photonics; http://www.hamamatsu.com/jp/ja/index.html). Autodeblur v 9.1 software (AutoQuant Imaging; http://www.meyerinst.com/index.htm) was used for 2D blind deconvolution. Stably transformed BY-2 cells were examined under a confocal laser scanning microscope (Leica TCS SP5 equipped with HyD detector; http://www.leica-microsystems.com).

### Transformation of by-2 cells with HR_1−_141T_908−_1053-E

A cDNA fragment for the 141 N-terminal amino acids was ligated to a region containing NLSm3 and NLSm4 and terminated with EGFP (HR_1−141_T_908−1053_-E) before insertion into a binary vector, pBI121. BY-2 cells were transformed with the vector using an *Agrobacterium*-mediated transformation method. BY-2 cells expressing the fusion protein were then selected on an MS medium supplemented with 4 μM 2,4-D and 50 μg/ml kanamycin, and then maintained on an MS medium containing 2,4-D without any kanamycin. The cells were suspension-cultured when needed.

### Definition of domains

The complete sequence of DcNMCP1 was divided into head (amino acids 1–58), rod (amino acids 59–691), and tail (amino acids 692–1164) domains, based on prediction of the coiled-coil domain using an algorithm developed by Lupas, (1997) (http://www.york.ac.uk/depts/biol/units/coils/mstr2.html). The extent of the sequence used in the fusion protein was specified with subscripted numbers corresponding to the amino acid numbering in the full-length protein.

### Introduction of point mutations

To modify the NLSm3 domain between amino acids 908 and 1053 of DcNMCP1 by addition of alanine residues, mutations were introduced to the original cDNA sequence by two rounds of PCR using primers NLS3mt-F and NLS3mt-R (Supplemental table S2). The plasmid, including the DcNMCP1 cDNA, was used as a template. The first round of PCR consisted of two separate reactions. NLS3mt-F and NM1-GFP-R3 (3^′^-end primer for amino acids 908–1053 of DcNMCP1) were used for one PCR reaction, and NLS3mt-R and NM1-GFP-F3 (5^′^-end primer for amino acids 908–1053 of DcNMCP1) were used for the other PCR reaction. The resulting two products were purified, and an equimolar-mixed product was used as the template for the second round of PCR, which used primers NM1-GFP-F3 and NM1-GFP-R3. The resulting product was inserted into the *Stu*I cleavage site of pKD0330. Using a similar methodology, amino acids of NLSm4 (amino acids 1023–1026) and its neighboring sequence YNL (amino acids 1020–1022) were replaced with A. A mutation in NLSm4 was introduced using the primers NLS4mt-F and NLS4mt-R, and in YNL using YNLmt-F and YNLmt-R.

### Sequence analyses

Comparison of nucleotide or protein sequences to sequences in databases and calculation of the statistical significance of matches were carried out using the computer program BLAST from NCBI (http://blast.ncbi.nlm.nih.gov). The WoLF PSORT server (http://wolfpsort.org/) was used to identify nuclear transport information within the primary sequence of DcNMCP1. Multiple alignments and a phylogenetic analysis were executed using ClustalW (http://clustalw.ddbj.nig.ac.jp/top-j.html) or MAFFT (http://mafft.cbrc.jp) software.

### Conflict of interest statement

The authors declare that the research was conducted in the absence of any commercial or financial relationships that could be construed as a potential conflict of interest.
