# Supplementary material for: Localization of Daucus carota NMCP1 to the nuclear periphery: the role of the N-terminal region and an NLS-linked sequence motif, RYNLRR, in the tail domain
Source: Front Plant Sci. 2014 Feb 26;5:62. doi: 10.3389/fpls.2014.00062 (PMC3935212; doi:10.3389/fpls.2014.00062)
Supplement: Supplementary file 1 [file DataSheet1.PDF]

Supplemental Table S1 The set of primers used for amplification of DNA fragment derived from DcNMCP1, DcNMCP2, and GST.

Fusion protein	Forward primer x Reverse primer
NMCP1-EGFP	NM1-Head-F x NM1-Tail-R
$RT_{142-1164}$	NM1-GFP-F1 x NM1-Tail-R
HR_{1-325}	NM1-Head-F x NM1-GFP-R1
$RT_{609-1164}$	NM1-GFP-F2 x NM1-Tail-R
$RT_{609-958}$	NM1-GFP-F2 x NM1-GFP-R2
$T_{908-1053}$	NM1-GFP-F3 x NM1-GFP-R3
$T_{975-1053}$	NM1-GFP-F4 x NM1-GFP-R3
$HR_{1-738}T_{975-1053}$	NM1-Head-F x NM1-GFP-R4 [1]
$H_{1-58}T_{975-1053}$	NM1-Head-F x NM1-GFP-R5 [1]
$HR_{52-738}T_{975-1053}$	NM1-GFP-F5 x NM1-GFP-R4 [1]
$HR_{1-141}T_{975-1053}$	NM1-Head-F x NM1-GFP-R6 [1]
$NM2_{1-150}T_{975-1053}$	NM2-Head-F x NM2-GFP-R1 [1]
GST-EGFP	GST-Head-F x GST-Tail-R

[1]Primer sets used for the amplification of DNA fragments that is inserted in front of the sequence coding amino acid 975-1053 from DcNMCP1.
