# Supplementary material for: Localization of Daucus carota NMCP1 to the nuclear periphery: the role of the N-terminal region and an NLS-linked sequence motif, RYNLRR, in the tail domain
Source: Front Plant Sci. 2014 Feb 26;5:62. doi: 10.3389/fpls.2014.00062 (PMC3935212; doi:10.3389/fpls.2014.00062)
Supplement: Supplementary file 2 [file DataSheet2.PDF]

Supplemental Table S2 Sequence of primers used for amplification of DNA fragment derived from DcNMCP1, DcNMCP2, and GST.

Name	Sequence
NM1-Head-F	5'-ATGTTTACTCCACCAAAG-3'
NM1-GFP-F1	5'-ATGCGCTCAGATTATGCT-3'
NM1-GFP-F2	5'-ATGCTTAATGATTTTGAG-3'
NM1-GFP-F3	5'-CCTGGAAAGAGAGCCAAG-3'
NM1-GFP-F4	5'-CCTTCACAGCCCTCTCCA-3'
NM1-GFP-F5	5'-ATGGGTTTGAATGCTAAATTG-3'
NM1-Tail-R	5'-tccacctccacctccAGTTGTGAGGAACTTCCA-3' [1]
NM1-GFP-R1	5'-tccacctccaccTTCAGTCAGATCTTTCTC-3' [1]
NM1-GFP-R2	5'-tccacctccacctccCCTGCTCTCATTAGT-3' [1]
NM1-GFP-R3	5'-tccacctccacctccACCACCATCATCAAT-3' [1]
NM1-GFP-R4	5'-tccacctccaccCCCACAATTCTTGCAGCT-3' [1]
NM1-GFP-R5	5'-acctccaccCAATTTAGCATTCAAACC-3' [1]
NM1-GFP-R6	5'-tccacctccaccGTCACGCAAGGCCTTCTC-3' [1]
NM1-NLS3mt-F	5'-GGACGCCAGgcGgcGgcGgcTgcAGTTGTTCCAGCAGTA-3' [2]
NM1-NLS3mt-R	5'-TGGAACAACTgcAgcCgcCgcCgcCTGGCGTCCTCCAGC-3' [2]
NM1-NLS4mt-F	5'-TACAACCTAgcGgcAgcCgcAACTGCAGCTCCGCTG-3' [2]
NM1-NLS4mt-R	5'-AGCTGCAGTTgcGgcTgcCgcTAGGTTGTACCGACC-3' [2]
NM1-YNLmt-F	5'-GCTCCAACTGGAgcGgcCgcCCTAAGGCGACACAAA-3' [2]
NM1-YNLmt-R	5'-GTGTCGCCTTAGGgcGgcCgcsTCCAGTTGGAGCCTG-3' [2]
NM2-Head-F	5'-ATGGCGAGTCCTCGATTA-3'
NM2-Head-R	5'-tccacctccaccGACTTTCGTTTCAGC-3' [1]
GST-Head-F	5'-CCTATGTCCCCTATACTAGGT-3'
GST-Tail-R	5'-acctccaccATCCGATTTTGGAGGATG-3' [1]
NM1-GST-F	5'-GATGTTTACTCCACCAAAG-3'
NM1-GST-R	5'-GGACCACCACCATCATCAAT-3'

[1]Lowercase letters indicate glycine-coding sequences.

[2]Lowercase letters indicate the changed bases
