# Supplementary material for: Localization of Daucus carota NMCP1 to the nuclear periphery: the role of the N-terminal region and an NLS-linked sequence motif, RYNLRR, in the tail domain
Source: Front Plant Sci. 2014 Feb 26;5:62. doi: 10.3389/fpls.2014.00062 (PMC3935212; doi:10.3389/fpls.2014.00062)
Supplement: Supplementary file 3 [file DataSheet3.PDF]

Supplemental Table S3 Accession numbers and other tags of the sequences shown in the Figure 5.

Name	Species	Accession number or ID
Dc1	*Daucus carota*	D64087
Ag	*Apium graveolens*	AB514506
Rc1	*Ricinus communis*	XM_002525923
Jc	*Jatropha curcas*	GAHK01012715
Pt	*Populus trichocape*	XM_002329281
Vv1	*Vitis vinifera*	CAO49297
Cs	*Cucumis sativus*	XM_004141446
Gm1	*Glycine max*	XM_003552589
Lj	*Lotus japonicas*	Chr1.LjB10L14.40.nc [1)]
Ca	*Cicer arietinum*	XM_004514352
Vv2	*Vitis vinifera*	CAN74873
Rc2	*Ricinus communis*	XM_002524342
At1 (LINC1)	*Arabidopsis thaliana*	NM_105392
Cas	*Camelina sativa*	GABL01088611
Sl	*Solanum lycopersicum*	XM_004234639
Gm2	*Glycine max*	XM_003520006
Dc3	*Daucus carota*	AB812877
At2 (LINC2)	*Arabidospsis thalianae*	NM_101194
Os	*Oryza sativa*	AB110204
Bd	*Brochypodium distachyon*	XM_003570165
Ta	*Triticum aestivum*	AK333905
Ac	*Allium cepa*	AB673103
Pp1	*Physcomitrella patens*	Pp1s76_81V6.1[2)]
Pp2	*Physcomitrella patens*	Pp1s200_64V6[2)]

1) Gene ID in miyakogusa.jp (http://www.kazusa.or.jp/lotus/index.html)

2) Transcript name in Phytozome (http://www.phytozome.net)
